# Osteosarcoma: prognosis plateau warrants retinoblastoma pathway targeted therapy

**DOI:** 10.1038/sigtrans.2016.1

**Published:** 2016-03-25

**Authors:** Sarah E Ballatori, Philip W Hinds

**Affiliations:** 1 Department of Developmental, Molecular and Chemical Biology, Tufts University School of Medicine, Boston, MA, USA

## Abstract

Osteosarcoma (OS) is the most common primary bone cancer in children and adolescents, affecting ~560 young patients in the United States annually. The term OS describes a diverse array of subtypes with varying prognoses, but the majority of tumors are high grade and aggressive. Perhaps because the true etiology of these aggressive tumors remains unknown, advances in OS treatment have reached a discouraging plateau, with only incremental improvements over the past 40 years. Thus, research surrounding the pathogenesis of OS is essential, as it promises to unveil novel therapeutic targets that can attack tumor cells with greater specificity and lower toxicity. Among the candidate molecular targets in OS, the retinoblastoma (RB) pathway demonstrates the highest frequency of inactivation and thus represents a particularly promising avenue for molecular targeted therapy. This review examines the present thinking and practices in OS treatment and specifically highlights the relevance of the RB pathway in osteosarcomagenesis. Through further investigation into RB pathway-related novel therapeutic targets, we believe that a near-term breakthrough in improved OS prognosis is possible.

## Introduction

Osteosarcoma (OS), also known as osteogenic sarcoma, is a malignant tumor of bone. It is the most common primary bone malignancy in children and adolescents, affecting ~560 young patients in the United States each year. Although OS is predominantly thought of as a disease of young adults, it typically impacts two age groups: those in the second decade of life and the elderly.^1^ More specifically, analysis of OS incidence from a long-term National Institute of Health study involving 3482 patients reaffirmed the bimodal age distribution, citing peak frequencies at age 15 and age 75. Patients under the age of 24 presented with predominately primary OS, with an incidence of 4.4 per million in this age group. The majority of the elderly patients presented with secondary OS, likely due to preexisting bone disorders. In addition, OS appears to occur more frequently in males than in females.^2^ Worldwide, OS incidence rates between different countries are generally consistent among individuals under 24 years old. Greater variations in the international OS incidence rates were observed in the elderly.^3^

The etiology of OS is elusive. Unlike Ewing’s sarcoma, which is another bone sarcoma that displays consistent genetic alterations in the form of chromosomal translocations, OS tumors are genetically diverse.^4^ Yet, there must be a molecular foundation in OSgenesis because several genetic diseases such as inherited retinoblastoma (RB), Li-Fraumeni syndrome and Rothman-Thomas syndrome demonstrate significantly increased incidences of OS.^5^ Moreover, evidence of OSs developing in siblings points toward a genetic origin.^6^ Etiological factors seem to be distinct for OSs in older patients, as these tumors develop and behave differently than those in younger patients.^5^ Thus, the etiology of OS is likely multifactorial and necessitates further research.

OS falls under the umbrella category of sarcomas, which are tumors of mesenchymal origin. More precisely and aligned with the cancer’s name, OS tumor cells characteristically produce immature osteoid and bone.^1^ All OSs are not created equally; the many different types of OS have varying associated prognoses. OS subtypes are classified based on multiple factors, including whether or not it originated from a preexisting lesion (primary versus secondary), location relative to the affected bone and histologic appearance. Conventional, telangiectatic, low grade and small cell comprise the intramedullary subtypes, while the surface subtypes include parosteal, periosteal and high grade.^7^ Conventional OS, the most common OS variant and the prototypical subtype for this paper, typically presents in the metaphyseal regions of long bones.^1,7^ About 40% of OSs present in the femur, 20% in the tibia, 10% in the humerus and 8% in the pelvis, with the additional cases dispersed throughout the skeleton and extremely rare cases presenting as extraosseous tumors.^8^

The standard treatment regimen is similar for the majority of OS subtypes. Treatment typically includes neoadjuvant chemotherapy to attack detected or presumed metastases, surgical resection with wide margins, and adjuvant chemotherapy. Advances in both surgical and chemotherapeutic protocols have improved patient outcome, as limb-preserving reconstructions have become standard practice and chemotherapeutics have increased the survival expectancy.^9^

Prognosis for conventional OS presenting without clinically detectable metastases is 70–80% survival at 5 years, and low-grade tumors are associated with an even better prognosis of 90% survival at 5 years.^8^ However, outcomes for patients who present with metastases drop to 20–40% survival at 5 years,^10^ which is also the case for patients presenting with skip metastases (small tumors within the intramedullary canal of the same bone).^11^ Pathologic fractures can also reduce survival expectancy to about 55% at 5 years.^12^ Finally, OS recurrence often results in pulmonary metastases and significantly decreases 5-year survival to about 15%.^13^ Interestingly, the interdisciplinary Cooperative German-Austrian-Swiss OS Study Group found that although 10-year survival for patients with extremity tumors reached nearly 70%, survival for patients with axial tumors remained below 33%.^14^ Perhaps these results are indicative of surgical advances in extremity OS. In general, low-grade lesions, negative surgical margins and >90% necrosis post-chemotherapy are indicative of a more positive prognosis, whereas axial tumors, large tumors, metastases, advanced patient age and secondary OSs represent negative prognostic factors.^8^

Despite the considerable efficacy of chemotherapy, the short- and long-term effects are severe. Treatment toxicity, in combination with the unfavorable prognosis for many patients, motivates researchers to invent new therapeutics with greater specificity and reduced toxicity. Continued translational research promises to achieve this, first by elucidating the molecular mechanisms at play in osteosarcomagenesis, and ultimately, through inspiring clinical trials of promising targeted therapeutics.^15^ Among the many molecular mechanisms implicated in osteosarcomagenesis, we believe that the RB pathway represents a critical field of research that promises to promote our understanding of OS and supply us with novel therapeutic targets.

## Treatment regimen

The most common treatment regimen today for patients with conventional OS includes neoadjuvant chemotherapy, wide surgical resection of the tumor and adjuvant chemotherapy. If the tumor is low-grade, chemotherapy is generally not needed. In addition, if pulmonary metastases are resectable, patients can undergo metastasectomies.^15^

Surgical resections must be skillfully and completely accomplished to ensure a favorable prognosis. Thus, surgeons must carefully evaluate the most recent images of the tumor site, incorporate the biopsy tract into the resection and remove the tumor safely within a layer of surrounding healthy tissue, also known as a wide margin. Using a wide margin for tumor removal controls local disease 95% of the time, which allows for surgical reconstruction.^8,9^ Beyond tumor removal, options for surgical resolution include amputation or, more commonly, limb preservation.^9^ Limb reconstructions are conducted with a variety of bone implants, such as endoprosthetic megaprostheses, bulk allografts or allograft prosthetic composites.^16–18^

The standard chemotherapeutic combination today includes methotrexate, doxorubicin, cisplatin and ifosfamide. Additional chemotherapeutic agents are occasionally added to the regimen, although none of these agents significantly alter the survival rates.^19^ In the majority of medical centers, chemotherapy is administered both before and after surgery despite evidence that preoperative chemotherapy does not affect disease-free survival.^20^ Neoadjuvant therapy’s widespread use is likely due to its benefits beyond survival, such as decreasing tumor size, eradicating micrometastases, enabling sufficient time for surgical planning, enabling delayed adjuvant treatment for postoperative healing and determining the extent of tumor necrosis.^9,21^ Knowing the degree of necrosis is particularly important for devising an effective adjuvant chemotherapy regimen and for more accurately predicting prognosis, as degree of necrosis correlates with an improved survival rate.^22^

Beyond the standard regimen of surgery and cytotoxic chemotherapy, targeted therapies are actively being pursued in translational research labs, clinical studies and even in approved treatments.^23^ For example, OS patients in Europe are currently being treated with immumostimulant mifamurtide in addition to the standard chemotherapy protocol. Although this drug is not currently approved in the United States, studies indicate that it can improve survival.^24^ Alternative attractive targeted therapies could employ monoclonal antibodies to specifically target cancer cells for destruction.^25^ In an effort to escape the four-decade-long survival rate plateau, many more novel therapies are under investigation, yet with limited success. We believe that the RB pathway holds significant promise to positively direct the evolution of OS treatment.

## Normal bone homeostasis

Prior to investigating any malignancy, it is important to appreciate that cancer results from the transformation of a normal cell. OS is no exception; tumor cells resemble mature osteoblasts, yet they are held in the immature osteoblast stage rather than fully differentiating into osteocytes. This implicates disruptions along the developmental process of osteoblastogenesis, several of which have already been characterized in OS.^26^ Thus, the search for novel therapeutics should begin with a strong understanding of the connections between the developmental generation of normal osteoblasts and the preservation or alteration of those processes in OS.

On a cellular level, bones are fashioned and maintained primarily by osteoclasts and osteoblasts. Osteoclasts are derived from precursor cells of the monocyte–macrophage lineage, which are in turn derived from the hematopoietic stem cell lineage.^27^ The process of osteoclastogenesis requires stromal cells and osteoblasts, as they produce receptor activator of nuclear factor kappa-B ligand and macrophage colony-stimulating factor. Those essential cytokines are released in membrane-bound and soluble forms by the stromal cells and osteoblasts.^28^ An additional important inhibitor of osteoclastogenesis, osteoprotegerin, serves as a decoy receptor for receptor activator of nuclear factor kappa-B ligand, thereby inhibiting it from binding the receptor activator of nuclear factor kappa-B receptor on osteoclast precursors.^28^

Osteoblasts derive from mesenchymal stem cells, which can produce fibrous connective tissue, fat, cartilage or bone. These cells are distinct from hematopoietic stem cells, which can ultimately produce osteoclasts.^29^ Osteoblastogenesis from mesenchymal stem cells requires transcription factors osterix and Runt-related transcription factor 2 (Runx2).^30^ Osterix, a zinc-finger-like transcription factor also known as SP7, is essential for bone formation, as studies have shown that osterix knockout mice develop skeletons with no bone, only cartilage. Osterix acts downstream of Runx2, as osterix is not expressed in Runx2 knockout mice, and Runx2 is expressed in osterix knockout mice.^31^ Runx2-null mice are also deficient in bone production, displaying a complete lack of mature osteoblasts. Runx2 is generally considered the key transcription factor for osteoblast differentiation, as it inspires the differentiation of mesenchymal progenitor cells to pre-osteoblasts.^32,33^ Furthermore, Runx2 is essential for bone sialoprotein and osteocalcin production.^30^ Studies have shown that RB protein (pRb) acts as a transcriptional coactivator of Runx2, contributing directly to the expression of osteoblast-specific genes, such as osteocalcin.^34^ Further investigation into the role of pRb in osteoblast differentiation will be covered in a later section.

Osteoblastogenesis continues as the osteoprogenitor cells start producing type I collagen, which provides bone with a strong structural framework for daily stresses and activity.^35^ Next, osteoprogenitor cells differentiate into alkaline phosphatase (ALP)-expressing pre-osteoblasts. ALP is an early differentiation marker, which is important for initiation of mineralization.^36^ Finally, pre-osteoblasts differentiate further into mature osteoblasts capable of synthesizing osteoid, the unmineralized, organic portion of bone matrix. With the addition of hydroxyapatite, the osteoid is mineralized and the osteoblast is encased in the matrix. In the final step of differentiation, the osteoblast becomes an osteocyte, a lining cell or undergoes apoptosis.^30^

Osteoblasts are remarkably diverse. This could explain the heterogeneity of bone microarchitecture throughout the skeleton, the propensity for OS to develop in certain bones more commonly than others, and the varied ability of osteoblasts to react to certain drugs. Unfortunately, this heterogeneity renders osteoblasts difficult to uniformly characterize genetically, likely contributing to the challenges facing OS research.^37^

However, appreciating osteoblast heterogeneity can be informative. For instance, OS is most commonly (>80%) found in the metaphyses of long bones, although it affects other bone types as well.^1^ Perhaps, because the metaphysis is the primary area of bone cell growth during childhood bone elongation, this region is more susceptible to OS development. This observation further underscores the importance of understanding how OS deviates from normal bone development.

## Elusive etiology

As briefly mentioned in the introduction, the true etiology of OS is a mystery. Countless studies have determined that OS’s etiology is multifactorial, including many genetic and environmental factors. The majority of cases appear to arise from sporadic, diverse mutations.^38^ Yet, it is clear that certain etiological factors, namely genetic diseases, may guide our studies toward more clarity. These genetic diseases indicate that molecular foundations for the development of OS indeed exist. OS incidence is increased in certain genetic diseases with tumor suppressor gene mutations, which are discussed below. Furthermore, Ottaviani and Jaffe^6^ have reported that, although rare, cases of siblings developing OS do exist.

Several genetic diseases have high incidences of OS, including inherited RB (defective RB (*RB1*) gene), Li-Fraumeni syndrome (inactive *p53* gene), and Rothman-Thomas, Werner and Bloom syndromes (DNA helicase abnormalities). Interestingly, OS in adults seems to develop and behave differently than in children and adolescents, suggesting separate etiological factors.^5^ The multitude of etiological factors in OS is perplexing, yet appreciating each candidate pathway will bring us closer to a cure.

Inherited RB predisposes primarily young children to both RB and OS. In this genetic disease, the *RB1* gene, which is located on chromosome 13q14.2, is defective, resulting in tumors of the retina.^39^ These tumors likely arise because the pRb encoded by *RB1* ordinarily functions in regulating cell proliferation and differentiation. Without functional pRb, regulation of the G1 to S phase transition of the cell cycle is lost, leading to unrestricted cell proliferation. Unfortunately, patients with inherited RB have a significantly increased risk of developing OS compared with the general population.^5^ Beyond inherited RB, *RB1* gene mutations have also been demonstrated in sporadic cases of OS.^38^

Li-Fraumeni syndrome also predisposes patients to OS at an alarming rate. This condition is characterized by autosomal dominant inheritance, early onset and a diverse array of cancers, including sarcomas, brain tumors, leukemias, adrenocortical tumors and breast cancer.^40^ Similar to inherited RB, the mutation in Li-Fraumeni syndrome results in the inactivation of a tumor suppressor gene (*p53*). Functional p53 is responsible for regulating cell cycle progression in the setting of DNA damage. Additional mutations of genes involved in the p53 pathway, such as p14ARF and MDM2, have been implicated in OS.^4^

A third class of genetic defects associated with increased OS incidence includes three DNA helicase abnormality syndromes: Rothman-Thomas syndrome, Werner syndrome, and Bloom syndrome. Rothman-Thomas syndrome is associated with short stature, cataracts, alopecia, skin changes and OS due to a defective *RECQL4* gene. Werner syndrome presents with melanomas, soft tissue sarcomas and OSs due to a defective *WRN* gene. Finally, Bloom syndrome’s defective *BLM* gene causes predisposition to a variety of cancers at a young age. In each of these syndromes, the defective genes (*RECQL4*, *WRN* and *BLM*) encode DNA helicases.^5^ Perhaps defective DNA helicases lead to tumors due to disrupted regulation of DNA replication, disrupted regulation of homologous recombination, and overall genomic instability.

OS in the adult population behaves quite differently than pediatric OS. For instance, prognosis for adult OSs is generally much worse than pediatric cases.^8^ OS in patients over 40 years old often arises as a secondary OS due to Paget’s disease, a chronic disorder caused by excessive breakdown and formation of bone. In fact, a hereditary factor in Paget’s disease, mutation of the *SQSTM1* gene, which is involved in osteoclastogenesis, could explain the eventual development of OS.^5^ Many other benign bone conditions, such as osteomyelitis, fibrous dysplasia, enchondromas and bone infarcts, correlate with OS development.^41^

Beyond genetic and physiological etiological factors, environment can also predispose to OS. A strong correlation exists between high-dose therapeutic radiation and secondary OS development.^42^ This correlation has been documented in both Ewing’s sarcoma and hereditary RB patients treated with radiation for primary tumors.^42,43^ In the case of hereditary RB, previous radiation treatment increases the risk of OS development 406-fold.^43^

Many more candidate OS etiology theories exist than can be listed in this review. Even more perplexing than these diverse postulated etiologies are the prognosis discrepancies, such as the minority of unsuccessful primary appendicular OS cases in which patients undergo the standard treatment regimen, yet develop metastases. It is our hope that continued research efforts discover a common thread to understand OSgenesis. However, genomic analyses of tumor tissue have revealed that this field of research will remain challenging; genetic signatures both between patients and even within the same patient’s metastatic nodules are not consistent.^44^

Because OS etiology remains elusive, many candidate pathways are currently under investigation in laboratories and clinical trials around the world. In an excellent review article of targeted therapies currently under investigation by the Pediatric Preclinical Testing Program, supported by the National Cancer Institute, Sampson *et al.*^25^ summarizes the outcomes of preclinical experiments conducted over many years. The group found that many agents produced significant activity in preclinical OS models. Agents included the following: multi-tyrosine kinase inhibitors, mTOR inhibitors, aurora kinase A inhibitors, Akt/PKB inhibitors, cyclin-dependent kinase (CDK) inhibitors, MEK1/2 inhibitors, p53 inhibitors, centromere-associated protein E inhibitor, histone deacetylase (HDAC) inhibitors, second mitochondria-derived activator of caspases mimetics and some conventional chemotherapies.^25^ Despite these extensive efforts to improve patient outcomes, we have achieved minimal progress since the introduction of chemotherapy in the 1970s. We believe this progress plateau warrants a shift of focus toward an irrefutable driver mutation in the RB pathway.

## The RB pathway

Briefly, considerable evidence suggests that disturbances in the RB pathway are central to the pathogenesis of OS. In this section, we justify this statement focusing on the many known functions of the RB pathway and the various RB disturbances in OS. Finally, we propose three ideas for targeted therapy.

### Background

pRb, a tumor suppressor, was initially identified due to its involvement in RB, a rare pediatric eye tumor. As previously mentioned, patients with RB have a defective *RB1* gene, which is located on chromosome 13q14.2.^39^ The *RB1* gene product, pRb, is a member of the pocket protein family and has been described to hold many roles, acting as a regulator of cell cycle progression, differentiation, developmental signaling, senescence and genomic stability. However, the characteristic and most thoroughly investigated role of pRb is its regulation of cell cycle progression via repression of E2F transcription factors.^45^ Evidence for pRb checkpoint regulation was demonstrated by unphosphorylated pRb microinjection into cells either early in G1 phase or late in G1/early S phase. pRb addition early in G1 resulted in G1 arrest, while pRb addition in late G1/early S phase resulted in no cell cycle arrest, allowing cells to progress to DNA synthesis and division.^45^

The Rb protein’s ability to regulate the G1/S checkpoint is modulated by phosphorylation. Studies have demonstrated that predominantly hyperphosphorylated pRb appears from late G1 through S, G2 and M phase. Predominantly hypophosphorylated pRb presents in early G1 and M phase.^46^ This cell cycle-dependent phosphorylation is facilitated by cyclins and CDKs. First, mitogen-dependent accumulation of cyclin D triggers CDK4 and CDK6 to phosphorylate pRb, which renders the pRb protein no longer functional as an E2F repressor. Free E2Fs can then promote transcription of genes required for DNA synthesis, along with additional cyclins E and A. Finally, cyclin E triggers CDK2 to continue pRb phosphorylation.^47^

In addition to E2F, Rb also interacts with HDACs and nucleosome remodeling complexes (SWI/SNF). pRb can form two unique complexes with these proteins, forming HDAC-pRb-SWI/SNF during G1 phase and pRb-SWI/SNF during S phase. Both of these complexes appear to function in the repression of E2F target genes, with HDAC-pRb-SWI/SNF repressing cyclin E and pRb-SWI/SNF repressing cyclin A and cdc2. As these pRb complexes appear to regulate the order of cyclin E and A expression, thus regulating exit from G1 and S phases, respectively, it is clear that the Rb protein’s regulatory power extends far beyond physical E2F repression.^48^

The pRb protein also has family members, p107 and p130, which have distinct yet largely overlapping roles with pRb. For instance, p107 and p130 can also repress E2F family transcription factors, recruit HDACs and other transcriptional repressors and regulate growth arrest. However, pRb and its related proteins display distinct roles in cell cycle regulation, as pRb, p130 and p107 each bind to different E2F family members. Furthermore, studies have revealed that mice with heterozygous pRb mutations develop tumors, while mice with p107 or p130 mutations are tumor free. Trouble arises only when mice have homozygous mutations for both p107 and p130, resulting in perinatal death. Thus, it appears that these pRb-related proteins cannot rescue the phenotype resulting from RB1 disruption in the mouse, yet they can functionally substitute for each other.^49^

A final component of the RB pathway, anti-mitogenic signals, serves to promote pRb regulatory function. Specifically, there are two major classes of CDK inhibitors, including INK4 and Cip/Kip proteins. The INK4 family, which inhibits CDK4 and CDK6, includes p16INK4a, p15INK4b, p18INK4c and p19INK4d. The Cip/Kip family, which inhibits CDK2, includes p21Cip1, p27Kip1 and p57Kip2. By inhibiting CDK function, these proteins help to maintain pRb in the hypophosphorylated state, thus enabling Rb to regulate cell cycle progression. Unfortunately, p16INK4a is often defective in human cancers, including OS.^49^

### Why focus on RB?

Synthesizing reports regarding the genetics and epigenetics of OS, we find that virtually all cases of OS display defects in the RB pathway.^38,50^ RB pathway abnormalities, discussed in a later section, include the following: defects in pRb, p16INK4a, CDK4 or cyclin D, along with epigenetic modifications of RB pathway genes. Beyond the sheer frequency of RB abnormalities observed in OS cases, there are many more equally compelling reasons to focus on this field.

For example, patients with germline mutations in the *RB1* gene (hereditary RB) have a 69-fold increased risk of developing OS compared with the general population. Their risk increases 406-fold with previous radiation therapy to the primary tumor site.^43^ Patients with sporadic RB have a much lower risk than patients with germline mutations. Furthermore, most sporadic OS cases have disturbances in the RB pathway. Taken together, these facts implicate RB as a driver mutation, having a central role in the pathogenesis of OS.^5^

Interestingly, effects of *RB1* inactivation are species, tissue and cell type specific. Mice with heterozygous *RB1* mutations develop predominantly pituitary and thyroid cancers.^51^ In the case of human hereditary RB, OS is the second most common cancer after RB itself. Moreover, although the RB pathway is the most commonly inactivated mechanism in human cancers, it appears that defects in individual members of the RB pathway result in distinctive tumor types. Overexpression of cyclin D1 predominates in breast cancer, while loss of p16INK4a predominates in melanoma. The *RB1* gene itself is most commonly targeted for inactivation in a specific subset of human tumors, including RB, OS, small cell lung carcinoma and bladder carcinoma.^52^ Based on these observations, we infer that pRb has a tissue-specific function in bone that is essential for tumor suppression.

In OS, degree of tumor differentiation, which holds powerful prognostic significance, demonstrates an inverse correlation with *RB1* loss. This means that prognosis worsens with increased *RB1* loss.^52^ It is clear that disruptions in osteoblast differentiation promote this malignancy, as mice with *RB1* driver mutations exclusively within the osteoblast lineage develop OS.^26^

The RB pathway must not be overlooked in the field of OS research. The pathway’s responsibilities in the cell cycle, differentiation, senescence and genomic stability need to be further characterized in order to design targeted therapeutics for OS. In this section, we discuss some of pRb’s primary responsibilities to illustrate the importance of this protein.

#### Mesenchymal differentiation

The pocket proteins mediate the differentiation of mesenchymal lineages into chondrocytes,^53^ myocytes^54^ and adipocytes.^55^ For example, experimental evidence indicates that loss of pRb disrupts myogenesis, as cell cycle control is essential for proper myoblast differentiation. Transcription factor MyoD provides the link between pRb and myogenesis, as it can both interact with pRb and regulate CDK function.^56^ In fact, MyoD can upregulate expression of CDK inhibitor p21CIP1, thus inhibiting CDK2 function and maintaining pRb in the hypophosphorylated, active state.^57^ Thus, MyoD both inspires myogenic phenotype and promotes cell cycle arrest, which highlights the interrelated nature of differentiation and growth arrest.

#### Osteoblastogenesis

Several studies have demonstrated that the pRb protein is essential for osteoblastogenesis. For instance, Feuerbach *et al.*^58^ employed a temperature-sensitive mutant of the simian virus 40-derived large T antigen, which ablates pocket protein function when active (at 33 °C) but does not when inactive (39 °C). Stromal osteoblast progenitor cells expressing this mutant oncogene could not differentiate at 33 °C, but they could at 39 °C. In other words, these progenitor cells could differentiate only when pocket proteins were functional.^58^ A second study further characterized the pocket proteins instrumental in osteoblast differentiation using both wild-type and mutant forms of the adenoviral oncoprotein E1A-12S. The wild-type 12S product inactivates both the pRb family and the p300/CBP family, while the mutant targets only p300/CBP. This study demonstrated that MC3T3-E1 preosteoblasts expressing the wild-type 12S did not differentiate into osteoblasts, while those expressing the mutant 12S could differentiate, implicating the pRb family in osteoblastogenesis.^59^

Thomas *et al.*^60^ further characterized the role of pRb in osteoblast differentiation, noting that pRb, but not p107 or p130, is essential for murine embryo fibroblasts (MEFs) to undergo bone morphogenetic protein 2 (BMP-2)-induced differentiation. BMP-2 treatment, which ordinarily promotes Runx2, ALP, osteocalcin and mineralization, stimulated the expression of these genes and processes in wild-type MEFs and in p107- and p130-deficient MEFs, yet RB^−/−^ MEFs failed to express late markers of differentiation, osteocalcin and mineralization. These results implicate pRb specifically in the regulation of terminal differentiation.^60^

To explain these findings, our lab investigated the link between pRb and Runx2, the key transcriptional regulator of bone formation. As previously mentioned, the Runx2 transcription factor is essential for osteoblast formation; mice lacking Runx2 display no osteoblast differentiation and die quickly after birth.^61^ In addition, Runx2 is essential for osteocalcin production.^30^ It is noteworthy that, in the previously mentioned Thomas *et al.*^34^ experiment, RB^−/−^ MEFs demonstrated Runx2 expression, yet they did not produce osteocalcin. The fact that Runx2 expression is not dependent on pRb, yet osteocalcin production is, suggests that the defective terminal differentiation in RB^−/−^ MEFs is downstream of Runx2 induction.^34^

Finally, an explanation for this relationship between pRb and Runx2 was established: pRb acts as a transcriptional co-activator for Runx2, promoting late osteoblast differentiation genes such as osteocalcin. In support of this theory, a physical association was discovered between pRb and Runx2 both *in vitro* and *in vivo*. This physical connection is mediated by each protein’s C-terminal domain, and it is that domain on pRb that is targeted for mutation in SAOS2 cells (a primary OS cell line). Related proteins p107 and p130, however, do not physically associate with Runx2. Beyond evidence of a physical interaction, chromatin immunoprecipitation studies have demonstrated that pRb’s ability to associate with osteoblast-related promoters is dependent on Runx2 expression. Furthermore, evaluating transcription levels at the native osteocalcin promoter in cells with dysfunctional Runx2, dysfunctional pRb or functional Runx2 and pRb, demonstrated that cells with functional Runx2 and pRb had double the transcriptional activity compared with Runx2 alone. Cell lines with functional pRb alone failed to initiate transcription. In addition, growth arrest is not sufficient to increase Runx2 transcriptional activity, as p107 and p130 could induce growth arrest in SAOS2 cells, yet no changes in Runx2 activity were observed. Taken together, the experimental evidence establishing cellular dependence of osteocalcin expression on pRb, a physical interaction between Runx2 and pRb, the recruitment of pRb by Runx2 to osteoblast-related promoters, and the doubled osteocalcin transcriptional activity observed with both pRb and Runx2 over Runx2 alone suggest that pRb acts as a transcriptional co-activator for Runx2.^34^

Interestingly, this mechanism is affirmed by analyses of human tumor samples. OS cells lacking pRb display markers of early osteoblast differentiation, including high levels of ALP. However, osteocalcin expression levels in these samples are minimal or absent, suggesting impairment in late stages of differentiation.^34,62^

#### Senescence

Nearly all cell types participate in the process of senescence, which has been demonstrated in primary cell cultures from a variety of species.^63^ Although the ticking of the molecular clock is classically attributed to the progressive shortening of telomeres, the RB pathway, specifically p16INK4a, is independently essential.^64^ Studying senescence with a focus on telomere maintenance has shed light on the specific role of the RB pathway in proliferation cessation.

It is well established that p16INK4a, along with other cell cycle inhibitors like p53 and p21CIP1, is upregulated in senescent cells. Continued cell division leads to the gradual accumulation of these cell cycle inhibitors, which effectively counts the number of cell divisions and leads to an irreversible G1 growth arrest.^65^ This counting mechanism is akin to the senescence regulating function of telomeres.

Telomerase lengthens telomeres, thus preventing the natural incremental shortening of telomeres and immortalizing cells. Overexpression of hTERT, a catalytic subunit of telomerase, can render cells immortal.^66^ Based on these observations, it follows that hTERT expression is increased in tumor cells, while its expression is undetectable in normal, somatic, non-malignant cells.^67^ To connect this information with the RB pathway, these hTERT-transduced immortal cells require either oncogene-induced or spontaneous disruption of the RB pathway, implicating RB as a collaborative yet separate senescence instigator.^34^ Oncogenic stimulation, which results in premature senescence, further underscores the RB pathway’s role in telomere-independent senescence. The constitutively activated small GTPase Ras causes cell proliferation followed by premature senescence in human diploid fibroblasts, yet ablation of pRb function allows these cells to bypass senescence.^68^ CDK inhibitor p16INK4a is similarly instrumental for senescence, as cells with inactive p16INK4a can also bypass Ras-induced senescence. In other words, the RB pathway has a critical role in premature senescence.^64,69^ Furthermore, the fact that immortalization of human cells necessitates RB pathway inactivation directly implicates RB dysfunction in development of the malignant phenotype. Gathering a better understanding of senescence induction is paramount, as the senescent phenotype is by its very nature anti-oncogenic.

#### Genomic stability

Genomic instability is a hallmark of OS. Specifically, OS demonstrates high levels of chromosomal instability (CIN).^70^ This means that OS cells often suffer duplication or deletion of whole chromosomes or parts of chromosomes.^71^ Although the cause of CIN is still in question, the primary suspects are dysfunctional mitotic checkpoint genes, such as RB. Experimental evidence supports this theory, as inactivation of the pRb protein results in CIN *in vivo*.^72^ In addition, *RB1* mutation leads to loss of heterozygosity and mitotic missegregation in mice.^38^ Regardless of the cause, CIN seems to be important for OS pathogenesis, as it leads to aberrations and variations among tumor cells.^72^

### RB mutations in OS

Disturbances in the RB pathway’s key players, including pRb, p16INK4a, cyclin D and CDK4/6, have been detected in OS tumors. pRb, which actively suppresses cell cycle progression when hypophosphorylated, is phosphorylated by the joint effort of CDK4/6 and cyclin D. p16INK4a inhibits CDK4/6, thus maintaining pRb in the hypophosphorylated, active state. Thus, functional pRb and p16INK4a can serve as tumor suppressors, while cyclin D and CDK4/6 promote proliferation. Loss of function mutations in pRb or p16INK4a, and amplification of cyclin D or CDK4/6 can lead to tumorigenesis.^38^ In this section, we will describe these aberrations in greater detail.

#### RB protein

Primary OS tumors often present with point mutations, deletions, or structural rearrangements in the *RB1* gene, located on chromosome 13q14.2. For example, 25–35% of sporadic OS cases display *RB1* mutations. 19–67% of sporadic cases display either loss of heterozygosity or deletion of the *RB1* locus. Not including hereditary RB cases, *RB1* expression is inactivated in about 50% of OS tumors.^38^

#### p16INK4a

A number of tumor types display p16INK4a mutations, such as bladder carcinoma, pancreatic adenocarcinoma, non-small cell lung cancer, melanoma, glioblastoma, oropharyngeal cancer and OS.^5^ Specifically in OS, p16INK4a inactivation is observed in about 10% of tumors.^52^ The majority of p16INK4a alterations are deletions rather than point mutations. The *CDKN2a* gene, which encodes both p16INK4a and p14ARF, is located on chromosome 9p21. Interestingly, the p14ARF protein regulates tumor suppressor p53 and is also commonly mutated in OS.^5^

#### CDK4 and cyclin D

About 10% of sporadic tumors display amplification of the *CDK4* gene, located on chromosome 12q13–14.^38^ Cyclin D is amplified in about 4% of OS cases. Of note, one study of 87 OS cases revealed that all samples with *CDK4* amplifications did not display INK4a disturbances, and *vice versa*.^5^ This indicates that there may be minimal overlap in RB pathway alterations. An additional gene that promotes cell cycle progression is also located at chromosome 12q13; DNA primase gene *PRIM1* is amplified in 42% of OS tumors.^38^

#### Epigenetic

Mutations of the RB pathway genes are not the only sources of RB dysfunction. Epigenetic alterations, especially DNA methylation, can lead to OS as well. In fact, epigenetic changes are incredibly common in all human neoplasia.^50^ DNA methylation is actually a normal occurrence through which gene expression is silenced starting early in development. This process is regulated by DNA methyltransferases, which add methyl groups to cytosine DNA nucleotides, and demethylases, which remove the methyl groups. These methylations typically occur in CpG dinucleotides, which are found with high frequency in promoters of normal genes. In normal genes that are destined for gene expression, the CpG islands are ordinarily unmethylated. As DNA methylation is normally a carefully orchestrated regulatory mechanism, methylation aberrations, such as promoter hypermethylation, can lead to genomic instability and tumorigenesis.^73^

*RB1* gene hypermethylation is a known contributor to the pathogenesis of many cancers, including hepatocellular carcinoma and RB.^51^ Through transcriptional silencing of tumor suppressor genes, methylation can effectively increase the rate of mutations, and both the mutations and DNA methylation are heritable.^50^ However, many experts feel that more definitive investigation into the role of methylation in OS tumorigenesis is necessary to reinforce its importance. Despite this, multiple analyses have revealed significant CpG methylation of the p16INK4a promoter (*CDKN2A* gene) in OS.^50,73^ Aberrant methylation is also significant in tumor suppressor gene *RASSF1A*, which has been shown to inhibit accumulation of cyclin D, thereby promoting cell cycle arrest.^50,74^ Thus, the observed hypermethylation of *CDKN2A* and *RASSF1A* in OS samples leads us to infer that methylation-induced inactivation of these tumor suppressor genes may have a role in osteosarcomagenesis.

Taken together, it becomes clear that at least one of these RB-related defects is likely to be apparent in each individual OS case. Perhaps an explanation for this lies in a longstanding scientific theory; *RB1* is a ‘gatekeeper’ gene whose inactivation enables premalignant cells to surpass a threshold, initiating tumorigenesis.^75^ Upon initiation of neoplastic progression, subsequent genetic alterations can accumulate and assist in tumor growth and metastasis.

### Ideas for targeted therapy

We believe that RB dysfunction is central to the pathogenesis of OS. As such, targeted therapeutics that functionally restore the RB pathway in malignant cells could have broad impact in most OSs. Unfortunately, it is naive to hope for literal restoration of the pRb protein in OS cells, so therapeutic avenues must instead compensate for each of pRb’s functions that are lost with *RB1* loss. We will discuss three potential therapeutic targets in this section, including one target for OS with functional pRb and two targets for OS without functional pRb.

#### Targeting mediators of tumor growth due to p16INK4a loss, or CDK4/6 or cyclin D amplification

**Figure 1:**
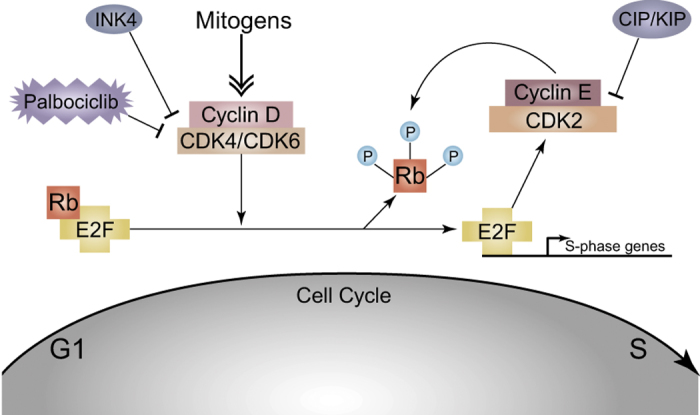
The classical pRb pathway demonstrates the utility of CDK inhibitors, such as palbociclib, in RB^+/+^ osteosarcoma. In this case, treatment of RB^+/+^ tumors with palbociclib would result in proliferation arrest and senescence as cells exit the cell cycle. In addition to its role as cell cycle regulator (pictured), pRb also serves as a transcriptional coactivator of Runx2 and thus promotes osteogenic differentiation (not pictured).

As previously described, p16INK4a serves as a tumor suppressor that inhibits CDK4/6 from phosphorylating pRb, and many OS tumors display p16INK4a loss, or CDK4/6 or cyclin D amplification. A natural resolution for this would be to employ CDK4/6 inhibitors, which could restore p16INK4a’s inhibitory function in cells. CDK4/6 inhibitors would also combat OSs displaying CDK4/6 or cyclin D amplification (Figure 1).

Compellingly, CDK4/6 inhibitors have recently shown promise in breast cancer trials. One such drug, produced by Pfizer (New York, NY, USA) and named palbociclib (Ibrance), selectively inhibits CDK4 and CDK6. This novel targeted therapeutic was approved by the Food and Drug Administration in February 2015 after experimental evidence revealed that it doubled progression-free survival in older women with advanced HER2-negative, estrogen receptor (ER)-positive (ER+) breast cancer. For now, palbociclib is used exclusively for this patient population, in combination with letrozole, as a first-line hormonal therapy. Of course, these tumors must also express functional pRb for palbociclib to have its effect. Two additional CDK4/6 inhibitors, LEE 001 from Novartis (Basel, Switzerland) and LY 2835219 from Eli Lilly (Indianapolis, IN, USA), are also under investigation for breast and other cancers.^76^ In October 2015, Lilly received Food Drug and Administration Breakthrough Therapy Designation for its CDK4/6 inhibitor, Abemaciclib.^77^

Similar to OS, breast cancer demonstrates dysregulation of the RB pathway, justifying the role of CDK inhibitors as novel breast cancer treatments. Overexpression of cyclin D1 (ref. 78) and alterations of p16INK4a^79^ are frequently found in breast cancer samples. Furthermore, hyperactivation of cyclin D1 and CDK4/6 are common specifically in ER+ breast cancer.^80^ ER+ breast cancer cells undergo growth arrest mediated by anti-estrogen therapy, which correlates with reduced cyclin D1 expression and thus pRb hypophosphorylation.^81^ Resistance to anti-estrogen therapy correlates with cyclin D1 overexpression, and thus pRb hyperphosphorylation and cell proliferation.^82^ Moreover, cyclin D1 can activate ER-mediated transcription, even in the absence of estrogen.^83^ These results provide strong evidence for CDK/cyclin/RB involvement in breast cancer and justify the use of this novel therapeutic (CDK4/6 inhibitor) in breast cancer patients, particularly those with ER+ cancer.^80^

Overall, clinical trials report that the side effects of palbociclib are predictable and manageable.^76^ In the PALOMA-1 palbociclib trial, the most common toxicity was neutropenia, which was observed in over 50% of the patients. Five patients discontinued the study, and about 40% of the patients needed to delay or reduce dosages due to toxicity. However, no infectious complications were observed. Additional side effects included nausea, diarrhea, alopecia and vomiting. Follow-up reports evaluating toxicity of palbociclib after 2 years found that its hematologic toxicity presents early in treatment, after which data do not support a cumulative, long-term hematologic toxicity.^80^ Of concern for palbociclib’s role in OS, studies have found that palbociclib may shield cancer cells from chemotherapy-mediated toxicity. This finding makes sense, as palbociclib serves to promote growth arrest in cancer cells, meanwhile cytotoxic chemotherapies generally target cells with rapid rates of proliferation. However, chemotherapeutics generally cause side effects that severely reduce quality of life, and the four-decade plateau in OS prognosis calls for novel targeted therapeutics. Ultimately, specific molecular targets for OS will allow doctors to stop prescribing toxic, non-specific chemotherapy regimens. Perhaps initial OS trials should evaluate the progression-free survival of RB+ patients treated with CDK4/6 inhibitors post-chemotherapy.^84^ This represents a novel therapeutic idea, as there are no listed clinical trials of CDK inhibitors in OS according to clinicaltrials.gov.

#### Targeting mediators of tumor growth due to RB loss—two ideas

The previously described targeted therapy effectively restores pRb function. However, when the pRb protein itself is lost due to genetic alterations, restoring its presence in cells is not an option. This poses a greater challenge for therapeutic strategies, as pRb holds many regulatory responsibilities; simply restoring the restriction on cell growth will not compensate for pRb loss. We propose two therapeutic avenues that have displayed effective pRb substitution in several studies.

#### KDM5A inhibition

KDM5A, also known as RB-binding protein 2 and JARID1A, is a histone demethylase. This protein catalyzes demethylation of dimethyl and trimethyl lysine 4 of histone H3 (H3K4me2 and H3K4me3), whose methylation is normally associated with transcriptionally active genes. Emerging evidence implicates KDM5A in human cancer pathogenesis, specifically via promotion of cell proliferation, repression of tumor suppressor gene expression, and development of drug tolerance.^85^ We believe that KDM5A inhibition is an excellent novel candidate for RB-negative OS therapy. Through a series of experiments, Benevolenskaya and several other scientists have elucidated the role of this protein, particularly in relation to pRb.

**Figure 2:**
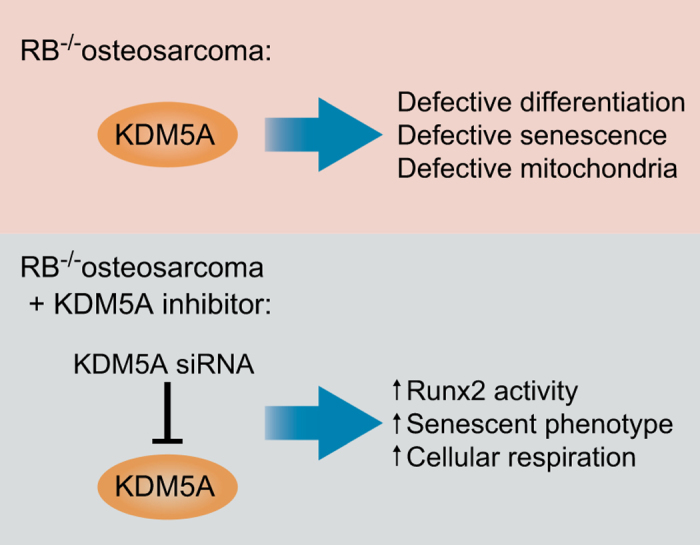
Key qualities of KDM5A underscore its therapeutic potential in osteosarcoma. Overall, KDM5A likely regulates osteogenic differentiation through repression of multiple different pathways of differentiation signaling. Thus, KDM5A inhibition is an excellent novel candidate for RB^−/−^ osteosarcoma targeted therapy.

Benevolenskaya *et al.* originally discovered KDM5A while screening for proteins that bind to pRb variants with impaired E2F binding. Subsequent investigation revealed that KDM5A and pRb colocalize, particularly in chromatin isolated from differentiating cells, and pRb’s capacity to promote differentiation correlated with binding of these two proteins. Importantly, post-transcriptional silencing of KDM5A via small interfering RNA (siRNA) restores pRb phenotype, which suggests that pRb binding to KDM5A inhibits KDM5A from repressing differentiation-specific genes. In other words, pRb promotes differentiation by inactivating KDM5A.^86^ Additional details from this study and others, described below, further support the concept of a KDM5A inhibitor for OS (Figure 2).

For example, studies have revealed that the cell cycle checkpoint function of pRb is distinct from its role in differentiation. pRb can become acetylated,^87^ which is not essential for the protein to bind with E2F, yet acetylation is essential for pRb to induce cellular differentiation.^88^ In addition, certain variants of pRb display defective E2F binding, yet they can promote differentiation and senescence.^89^ Accordingly, we can infer that the role of pRb in differentiation is E2F-independent. The previously mentioned pRb variants that lack pRb/E2F transcriptional repressor complexes, along with RB1-null cells transfected with KDM5A siRNA, ultimately stop proliferating.^86^ Thus, although KDM5A inhibition would not restore pRb’s repressive effect on E2F, it could still serve to cease cancer cell proliferation in RB mutants via induction of a differentiation-like state.

pRb seems to promote transcription of differentiation-specific genes with the help of differentiation-specific transcription factors, such as MyoD and Runx2.^34,86^ In RB1-null cells, KDM5A silencing with siRNA results in increased activity of these differentiation-specific transcription factors.^86^ Thus, pRb and KDM5A siRNA share the ability to promote these differentiation-inducing transcription factors, further supporting the idea of KDM5A inhibition in OS therapy. To further characterize the relationship between pRb, KDM5A and cell differentiation, Benevolenskaya *et al.*^91^ demonstrated that loss of KDM5A restores differentiation in RB^−/−^ cells via increasing mitochondrial respiration. RB^−/−^ cells exhibit defective mitochondria and decreased oxygen consumption, while KDM5A is a direct repressor of metabolic regulatory genes. In addition, mitochondrial biogenesis regulator PPARGC1A, which activates mitochondrial function, inhibits RB^−/−^ cancer cell growth and thus overrides the differentiation block. Taken together, these findings implicate KDM5A in mitochondrial repression in RB^−/−^ cells and highlight the switch to oxidative phosphorylation as an essential mechanism in restoring cellular differentiation. KDM5A inhibition is therefore a logical therapeutic concept for RB^−/−^ OS, as its inhibition would restore mitochondrial function, allow for differentiation, and ultimately diminish tumor cell proliferation.^90,91^

Despite the restored pRb phenotype in RB-null cells with KDM5A siRNA, including the capacity to differentiate, there are additional facets of the differentiation pathway that may not be recovered in these cells. For instance, pRb may participate in KDM5A-independent activities, such as E2F regulation, that could contribute to successful differentiation. In addition, KDM5A actually cooperates with pRb on occasion, such as to activate transcription of homoeotic genes *BRD2* and *BRD8*. It is clear that KDM5A inhibition would not completely restore a normal phenotype in RB^−/−^ cancer cells. However, KDM5A inhibition seems to successfully promote cell cycle exit and at least partial differentiation. Perhaps the theoretical KDM5A-independent pathways to differentiation and the KDM5A transcriptional activation of homoeotic gene are not indispensable for successful differentiation.^86^

Experimental evidence strongly suggests that KDM5A inhibition specifically promotes osteogenic differentiation and senescence. For instance, Flowers *et al.*^92^ discovered that pRb directly activates the ALP promoter by displacing KDM5A. As ALP is an early marker of osteoblast differentiation, which is repressed by KDM5A, this finding further supports the utility of KDM5A silencing in RB^−/−^ OS.^92^ Ge *et al.*^93^ studied KDM5A knockdown in human adipose-derived stromal cells (hASCs) and further confirmed that KDM5A holds repressive power over osteogenic differentiation. In this study, KDM5A siRNA in hASCs promoted osteogenic differentiation both *in vitro* and *in vivo*. Moreover, KDM5A silencing caused significantly increased expression of osteoblast differentiation genes, including osterix, ALP and osteocalcin.^93^ KDM5A has been discovered at each of these gene’s promoters, likely to repress gene expression.^92,93^ Beyond promoter regulation, KDM5A has also been found to be both physically and functionally associated with Runx2.^93^ Thus, KDM5A likely regulates osteogenic differentiation at many levels through repression of multiple different pathways of differentiation signaling. Finally, the importance of KDM5A is also apparent in OS cells. Premature senescence can be induced in RB^−/−^ SAOS2 (primary OS) cell lines by reintroduction of pRb into the cells.^34^ Similarly, using KDM5A siRNA in these cells induces a senescent phenotype.^86^

Intriguingly, KDM5 family inhibition represents a second similar candidate therapeutic target for both ER+ breast cancer and OS. KDM5B is highly expressed in ER+ breast cancer samples. Furthermore, using KDM5B short hairpin RNA interference in MCF-7 cells (breast cancer cell line) causes a significant decrease in tumor growth.^94^ Perhaps the field of KDM5 in breast cancer will continue to inspire OS researchers, particularly if KDM5 inhibitors gain clinical approval. The universal importance of KDM5 proteins in human cancers is appreciable. Looking to the future, specific inhibitors for KDM5 must be developed to better understand this novel anticancer therapy.^85^

#### Hedgehog agonist

Similar to the RB pathway, the hedgehog (Hh) pathway is also associated with OS, and it is instrumental in osteoblast differentiation.^95^ The Hh pathway is a highly conserved developmental regulator, which includes a cascade of signaling components that collaborate to promote stem cell regulation, organogenesis and tissue regeneration. Hh signaling involves Hh ligands (sonic Hh, Indian Hh and Desert Hh), transmembrane receptor proteins Patched 1 and Patched 2 (PTCH1 and PTCH2), G protein-coupled receptor smoothened (SMO) and transcription factors (GLI1, GLI2 and GLI3). Indian Hh is of specific interest for this paper, as it regulates bone and cartilage development. Ordinarily, PTCH inhibits SMO, maintaining SMO at the base of primary cilia. When Hh ligand reaches the target cell, it binds PTCH to release SMO from PTCH-mediated inhibition. Free SMO can then translocate to the tips of the cilia, which activates GLI transcription factors to accumulate in the nucleus and regulate transcription of Hh target genes.^96^ Essentially, the secreted Hh protein serves as a morphogen, which diffuses down its concentration gradient to trigger various cellular responses.^97^

The link between the Hh and RB pathways was initially established through investigation of the transcription factor Runx2. As previously discussed, Runx2 is indispensable for osteoblastogenesis, and it interacts with other co-regulators, such as pRb, that influence transcription of its target genes.^60^ Runx2 holds a similar, inductive role in chondrogenesis, which correlates with its ability to directly induce Indian Hh (Ihh) expression.^61^ Runx2 directly binds to the promoter region of the *Ihh* gene and induces gene expression, as evidenced by reporter gene assays.^98^ As additional evidence, Runx2^−/−^ animals exhibit markedly decreased Ihh expression. Beyond Runx2 regulation of Ihh, it appears that Ihh can initiate a feed-forward loop in osteoblastogenesis, as Ihh has been shown to upregulate Runx2 via the Hh pathway’s GLI2 transcription factor in MEFs.^99^ Collectively, these data strongly implicate Ihh in regulation of osteoblast differentiation and establish a link between Ihh and pRb, as both proteins are intimately involved with Runx2. Our preliminary data, described below, support these assumptions, implicating Ihh as a key downstream target of pRb that may in turn explain the retention of both adipogenic and osteogenic differentiation programs in pRb-null murine osteoblasts.^100–104^

In an excellent review article of the Hh pathway, authors remind us that Hh signaling is incredibly complex. Despite active research accomplishments during the past two decades, there remain substantial gaps in our understanding of Hh signaling.^97^ The unanswered questions in the field of Hh signaling likely contribute to the present controversy regarding its role in OS.

Many studies have found that OS cell lines and OS biopsy samples overexpress components of the Hh pathway. Logically, inhibitors of this pathway, specifically smoothened antagonists, were tested in OS cell lines as a potential targeted therapy. For example, Hirotsu *et al.*^105^ postulate that SMO inactivation may be an effective therapeutic for patients with OS. In this study, cyclopamine was used to specifically inhibit SMO in OS cells *in vitro*, which resulted in slowed growth and apparent G1 arrest. In addition, SMO short hairpin RNA prevented OS growth *in vitro* and *in vivo*.^105^ In a different study, Lo *et al.*^106^ more extensively evaluated the possibility of employing Hh inhibitors as targeted therapeutics for OS. Overall, the investigation confirmed the complexity of Hh signaling, noting that OS cells lines exhibit both ligand-dependent and ligand-independent activation of Hh target genes. The ligand-independent cells overexpressed GLI2, which could only be reduced using GLI inhibition. On the other hand, overexpression of Hh target genes in ligand-dependent cells could be regulated by both SMO and GLI. Furthermore, using SMO antagonist IPI-926 in two patient-derived, ligand-dependent xenograft models resulted in Hh signaling inhibition and antitumor function in only one of the two models. The authors conclude that SMO inhibitor IPI-926 warrants additional research, as it could potentially serve as an OS treatment option.^106^ Current literature continues to support the concept of Hh inhibition as a novel therapeutic target for OS.^107^

These data are consistent with Hh activity in other cancers, such as medulloblastoma, basal cell carcinoma, rhabdomyosarcoma and meningioma. These cancers can exhibit loss of function of PTCH or SUFU, or gain of function of SMO, which results in constitutively active ligand-independent Hh signaling. Specifically in basal cell carcinoma and medulloblastoma, SMO inhibitors have proven to be effective monotherapy options for those patients. Unfortunately, Hh inhibitors have demonstrated only limited efficacy in other cancers.^96^

On the other hand, many studies support the idea of Hh agonists as OS targeted therapeutics. For example, Jemtland *et al.*^108^ found that Ihh expression levels correlated with increasing osteoblast maturation. Furthermore, recombinant N-terminal sonic Hh, which upregulates Hh target genes, was shown to increase ALP expression and activity. Collectively, the data suggest that Hh signaling holds a functional role in osteoblast differentiation.^108^ Another study demonstrated that removal of SMO from perichondrial cells prevents both normal bone collar and primary spongiosa formation. In addition, SMO^−/−^ chimeric embryos failed to develop osteoblasts, but could generate chondrocytes. Also, BMP-induced osteogenesis in a limb-bud cell line required Hh signaling. This series of experiments reinforced the notion that Ihh is directly required for osteoblast development, specifically in long bones.^109^ A third study of note demonstrated that oxysterols, which are known inducers of osteoblastogenesis from pluripotent mesenchymal cells, promote osteogenesis by activating the Hh pathway through indirect activation of SMO.^110^ Fourth, investigating the ability of hASCs to heal critical-sized calvarial defects revealed that hASC-derived Hh signaling may promote skeletal repair via paracrine signaling to mouse calvarial osteoblasts. Analyzing the conditioned medium displayed a correlation between mouse calvarial osteoblast osteogenic differentiation and Hh signaling activation. Furthermore, the conditioned medium’s pro-osteogenic effect on mouse calvarial osteoblasts duplicated with the addition of Hh agonists (recombinant Shh and SMO agonist); however, SMO antagonist cyclopamine reversed the effect. Cyclopamine addition *in vivo* also impaired hASC-mediated bone repair.^111^ Most recently, Nakamura *et al.*^112^ identified novel Hh agonists (Hh-Ag 1.3 and 1.7) that activate GLI1 expression and induce mesenchymal stem cells to undergo osteoblast differentiation. Both Hh-Ag 1.3 and 1.7 displayed dose-dependent stimulation of ALP activity and induction of osteoblast-specific genes in the mesenchymal stem cell line C3H10T1/2. Interestingly, Hh-Ag 1.7 specifically induced osterix (downstream target of Runx2) expression, and it was able to rescue the osteoblast differentiation defect in a Runx2-deficient murine mesenchymal cell line.^112^

In summary, the Hh signaling pathway has been extensively investigated, yet controversy remains regarding its role in OS. These studies reinforce the Hh pathway’s important role in osteoblast differentiation. As osteoblast differentiation is often lost in OS tumors, novel therapeutics should aim to repair this process using Hh agonists.

The utility of Hh agonists is particularly important in RB^−/−^ OS. As previously discussed, a link between pRb and Ihh signaling was initially plausible due to Runx2’s ability to induce Ihh expression, and *vice versa*.^98,99^ Elucidation of the Ihh signaling pathway’s regulatory role in osteoblast differentiation represents a second, strong parallel between Ihh and pRb.^109,110^ Finally, our observations of dysregulated Ihh signaling in RB-null intestinal epithelial cells served as additional impetus to investigate the relevance of the Ihh pathway in RB deficient OS.^113^

**Figure 3:**
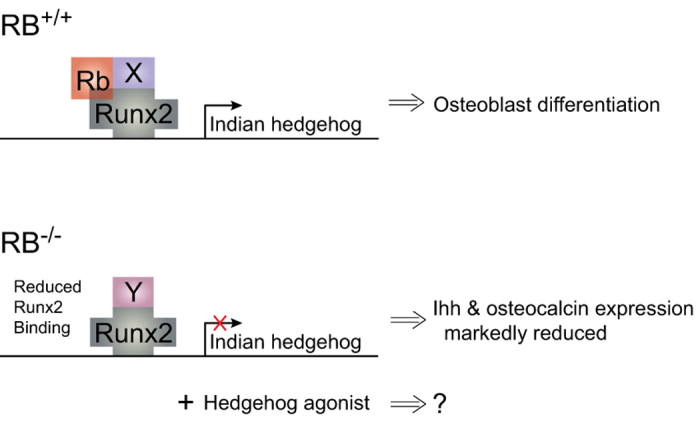
Indian hedgehog (*Ihh*) gene expression is markedly reduced in RB^−/−^ murine osteoblasts. In addition, expression of osteocalcin, another gene for which Runx2 serves as a transcription factor, is reduced in these cells. Chromatin immunoprecipitation analysis reveals that both Runx2 and pRb bind to the *Ihh* promoter in RB^+/+^ cells, while pRb is absent and Runx2 binding is significantly reduced in RB^−/−^ cells, despite equivalent Runx2 expression. This model posits a chromatin-modifying factor ‘X’ that is recruited to the Runx2 complex by pRb to positively activate *Ihh* transcription, and a factor ‘Y’ that may alternatively repress *Ihh* transcription when pRb is not present.

Preliminary studies in our lab indicate that *Ihh* expression in osteoblasts is indeed altered with RB deficiency. For example, examining *Ihh* expression in RB^−/−^ and wild-type calvarial cells revealed that *Ihh* expression is initiated during late osteoblast differentiation in wild-type cells, yet it is not in RB^−/−^ cells. Moreover, chromatin immunoprecipitation studies demonstrated that pRb is detected at the Ihh promoter in RB^+/+^ cells. pRb is nonexistent in RB^−/−^ cells, and, despite equivalent Runx2 expression in these cells, pRb loss correlates with reduced Runx2 binding to the *Ihh* promoter. Collectively, these preliminary data suggest that pRb acts as a transcriptional coactivator with Runx2 for genes other than *BGLAP* (encodes osteocalcin); together, pRb and Runx2 likely promote *Ihh* expression during late osteoblast differentiation.^100^ In light of these preliminary data implicating pRb as an inducer of Ihh signaling, coupled with the knowledge that Ihh signaling can positively influence osteoblast lineage commitment and maturation, we feel that promotion of the Ihh signaling pathway may serve as an excellent therapeutic avenue for RB-deficient OS. Specifically, SMO agonists must be further investigated to determine whether this novel therapeutic idea can mediate the consequences of pRb loss in OS (Figure 3).

### Conclusion

OS is complex. Challenges encumbering this field of research stem from the rarity of OS, the lack of pathognomonic mutations, and the genomic inconsistencies apparent both between and within individual tumors. Recent whole-genome sequencing studies have confirmed the genomic chaos characteristic of OS, citing high levels of CIN and *RB1* among the most commonly interrupted genes. These international sequencing efforts are increasing in number and scope in an effort to discover new therapeutic targets.^114^

Despite tremendous improvements in OS survival in the 1970s and 1980s, advances in OS treatment have reached a discouraging plateau over the past several decades. A major challenge facing OS treatment advancement is targeting metastatic, relapsed, and/ or refractory disease, for which prognosis has remained poor.^115^ For this reason, the notion of RB-targeted therapy is particularly promising, as it would continue to specifically target micrometastases formerly overlooked by conventional surgical resection and adjuvant chemotherapy.

In general, trials that specifically target RB-null cells are in the early, pre-clinical, stages. These studies employ a diverse array of molecular strategies, such as *TSC2* inactivation, suppression of glycolytic and glutaminolytic metabolism, and infection with oncolytic viruses.^116–118^ AdΔ24, a conditionally replicating adenovirus vector that contains a deletion in the E1A region to prevent pRb binding, has been investigated specifically in human RB-null OS cells.^118^ One example of a class of RB-targeted therapeutics in phase I clinical trials is the HDAC inhibitors, which function optimally in cells with elevated E2F1 activity and thus work best in RB-null cells.^119^ These examples, along with the multitude of studies not listed, make it clear that the field of RB targeting will continue to bolster momentum in order to better characterize and ultimately identify an optimal therapeutic target for RB-null malignancies.

Looking to the future, ideal OS therapeutics will aim to attack tumor cells with greater specificity and reduced toxicity. However, this quest for effective targeted therapeutics will remain challenging due to the elusive etiology of OS. We believe, for several reasons previously outlined in this paper, that RB pathway inactivation serves as an irrefutable driver mutation in osteosarcomagenesis. Because RB inactivation is so prominent and potent in OS tumor cells, RB-related therapeutics such as CDK inhibitors, KDM5A inhibitors and Hh agonists deserve the spotlight in the field of OS research.
